# Human epithelial-type ovarian tumour marker beta-2-microglobulin is regulated by the TGF-β signaling pathway

**DOI:** 10.1186/s12967-016-0832-x

**Published:** 2016-03-16

**Authors:** Wenwen Sun, Lu Gui, Xulei Zuo, Lingyun Zhang, Daibing Zhou, Xiaoling Duan, Weimin Ren, Guoxiong Xu

**Affiliations:** Center Laboratory, Jinshan Hospital, Fudan University, 1508 Longhang Road, Shanghai, 201508 People’s Republic of China; Department of Pathology, Jinshan Hospital, Fudan University, Shanghai, 201508 China; Department of Obstetrics and Gynecology, Jinshan Hospital, Fudan University, Shanghai, 201508 China; Department of Oncology, Shanghai Medical College, Fudan University, Shanghai, 200032 China

**Keywords:** B2M, Epithelial ovarian cancer, Tumorigenesis, TGF-β signaling, Biomarker, Therapeutic target

## Abstract

**Background:**

Beta-2-microglobulin (B2M), a light chain subunit of the major histocompatibility complex (MHC) class I complex, has been implicated in tumorigenesis. However, whether it is expressed in different epithelial-type ovarian tumours remains unknown. This study was performed to examine the expression of B2M in different histopathological types of ovarian tumours, to explore the function of B2M in ovarian cancer (OC) cells and to investigate the mechanisms underlying the regulation of B2M by the TGF-β signaling pathway.

**Methods:**

B2M expression in normal ovarian tissues and epithelia-type ovarian tumours was detected by immunohistochemistry and Western blot, followed by the analysis of association with clinical features. OC cells were transfected with B2M-siRNA and cell proliferation, migration and invasion were determined by WST-1 assay, wound healing assay and Transwell invasion assay, respectively. The regulation of B2M by the TGF-β signaling pathway in OC cells was examined by Western blot, ELISA and qRT-PCR.

**Results:**

We found that B2M was overexpressed in ovarian borderline and malignant tumours compared with benign tumours and normal controls, but was not associated with age, tumour size, lymph node metastasis and clinical stage. Knocking down of B2M led to a decrease in OC cell proliferation, migration and invasion. The expression of B2M was downregulated by TGF-β1 in OC cells, which was abolished in the presence of the inhibitor of TGF-β type I receptor.

**Conclusion:**

Our findings suggest that B2M is a potential tissue biomarker and therapeutic target of borderline and malignant ovarian tumours and the dysregulation of B2M in these tumours may be mediated by the TGF-β signaling pathway.

## Background

Beta-2-microglobulin (B2M), a light chain subunit of the major histocompatibility complex (MHC) class I complex, is associated with the heavy chain (α-chain) of the complex on the surface of nearly all nucleated cells. It exists in two forms, membrane and soluble B2M, which freely exchange with each other [1–3]. A high level of soluble B2M is detected in several types of cancers, including breast [4], colorectal [5], gastric [6], lung [7], oral [8], ovarian [9], prostate [10] and testicular [11] cancers, and may be implicated in the immune escape of cancer cells and cancer immunotherapy [12–15]. Accumulating data suggest that B2M may be a biomarker for cancers and a potential target for cancer therapy. The functional study of B2M in cancer cells has shown that B2M plays an important role in the promotion of cancer cell growth [16]. The growth-stimulatory activity of B2M may be associated with the antagonistic activity of B2M to the transforming growth factor-β (TGF-β)-induced growth inhibition [17].

TGF-β belongs to a superfamily of secreted cytokines and plays a pivotal role in immunosuppressive action. TGF-β signals through the activation of serine/threonine kinase receptors on the cell surface and their transducer proteins, Smads such as Smad2/3, in the cytosol [18, 19]. It has been shown that TGF-β signaling is dysregulated in several human diseases [20], including epithelial-type ovarian malignant tumour often referred to as epithelia ovarian cancer (EOC) which is the most lethal gynecological malignancy [21]. TGF-β modulates the invasion of ovarian cancer (OC) and affects the metastatic potential of OC cells [22–24]. Our previous study also shows that TGF-β1 can mediate a progression marker cystatin B in human epithelial-type ovarian tumours [25].

The present study was undertaken to examine the expression of B2M in human epithelial-type ovarian tumours, including benign, borderline and malignant tumours, and to investigate the biological function of B2M in OC cells. We further explore the mechanisms underlying the regulation of B2M by the TGF-β signaling pathway.

## Methods

### Patients and ovarian tissue sample preparation

All samples were obtained with informed consent from patients and the study on human subjects was approved by the Ethics Committee of Jinshan Hospital, Fudan University. A total of 148 paraffin-embedded ovarian samples were collected from patients (40 normal samples from patients with non-ovarian tumour and 108 epithelial-type tumour samples, including 38 benign, 38 borderline and 32 malignant tumours) with median age 50 years (range 17–81 years) at Jinshan Hospital, Fudan University (Shanghai, China) between 2005 and 2014. All patients underwent cytoreductive surgery. None of the patients had received a chemotherapy or radiotherapy before surgery. The 10 % formalin-fixed paraffin-embedded ovarian tissue specimens were prepared and pathologically diagnosed in the Department of Pathology. Four micrometer thick tissue of specimen was sectioned for the histological examination and the immunohistochemical (IHC) assay.

### Immunohistochemical staining and analysis

IHC analysis was performed as described previously [25]. Briefly, the sections were deparaffinized and rehydrated. After blocking with 10 % normal goat serum (Maixin Bio, Cat# SP KIT-B2, Fuzhou, Fujian, China) for 10 min at room temperature, the sections were incubated with a polyclonal rabbit anti-B2M antibody (1:1000 dilution, Abcam, Cat# ab15976, Cambridge, UK) at 4 °C overnight, followed by incubation with biotinylated anti-rabbit secondary antibody (Maixin Bio, Cat# KIT-9922) at room temperature for 1 h. After washing with PBS, the signal was detected using a DAB Kit (Maixin Bio). Finally, the sections were counterstained with hematoxylin and photographed under a light microscope (BX43, OLYMPUS, Tokyo, Japan). A brown color in epithelial cells was considered as positive staining. The evaluation of B2M immunoreactive staining was done by two independent pathologists without any prior knowledge of patient’s clinical data. The scoring system [25] was classified into B2M-positive and -negative groups. A normal ovarian tissue without a primary antibody was used as a negative control.

### Protein extraction from frozen ovarian tissue

The fresh tissue of the ovaries from patients were immediately immersed in liquid nitrogen after surgical resection and then stored at −80 °C until use. About 100 mg of tissue sample was lysed with 1 ml of ice-cold SDS lysis buffer (Beyotime, Haimen, Jiangsu, China) with 1 % PMSF (Beyotime) and 1 % phosphatase inhibitor (KeyGEN, Nanjing, Jiangsu, China), followed by dissociation using homogenizer. After centrifugation at 3000 rpm for 5 min, the supernatant was transferred to a new tube, followed by sonication for 15 s. Samples were then centrifuged at 14,000 rpm for 15 min at 4 °C. The pellets were discarded and the concentration of protein in supernatant was measured by a BCA Protein Assay kit (Thermo Scientific, Rockford, IL, USA). Lysates were then stored at −80 °C until use.

### Cell culture and TGF-β treatment

Human OC cell lines, OVCAR-3 and SK-OV-3, were purchased from American Type Culture Collection (ATCC). Cells were cultured as described previously [25]. For TGF-β1 treatment, cells were seeded for 24 h and then treated with 10 ng/ml recombinant human TGF-β1 (R&D Systems, Cat# 240-B, Minneapolis, MN, USA) for 24, 48 or 72 h. SB-431542, a TGF-β type I receptor kinase inhibitor, was obtained from Sigma (Cat# S4317-5MG, Saint Louis, MO, USA) and dissolved at a concentration of 10 mM in dimethyl sulfoxide (DMSO) (Sigma). For blocking the TGF-β signaling pathway, cells were pre-treated with 10 μM SB-431542 for 30 min and then treated with TGF-β1 for 48 or 72 h. Cells without any treatment were used as a control.

### Small interfering RNA transfection

OVCAR-3 and SK-OV-3 cells were plated into 6-well plate at a density of 2 × 10^5^/well and 1.5 × 10^5^/well, respectively, for 24 h and then transfected with 2 μg/well (OVCAR-3) or 1.5 μg/well (SK-OV-3) of human B2M-small interfering RNA (B2M-siRNA) or nonspecific scramble control siRNA (C-siRNA). The sequences of human B2M-siRNA were 5′-CCAGCGUACUCCAAAGAUUTT-3′ (sense) and 5′-AAUCUUUGGAGUACGCUGGTT-3′ (antisense) and the sequences of C-siRNA were 5′-UUCUCCGAACGUGUCACGUTT-3′ (sense) and 5′-ACGUGACACGUUCGGAGAATT-3′ (antisense) (synthesized by GenePharma, Shanghai, China). The siRNA was diluted in 100 μl serum-free Opti-MEM medium (Gibco, Cat# 31985-070, Thermo Fisher Scientific), mixed with 10 μl (OVCAR-3) or 8 μl (SK-OV-3) X-tremeGENE siRNA Transfection Reagent (Roche Diagnostics, Cat# 4476093001, Indianapolis, IN, USA) and incubated for 20 min at room temperature according to the manufacturer’s instruction. After transfection for 6 h, the medium was removed and replaced with fresh RPMI-1640 (OVCAR-3) or DMEM (SK-OV-3) supplemented with 10 % FBS without antibiotics. Cells were further incubated for the experiments as indicated.

### Cell proliferation assay

Cells were plated into a 96-well plate at a density of 4500/well (OVCAR-3) or 3000/well (SK-OV-3) and transfected with 0.6 μg/well (OVCAR-3) or 0.5 μg/well (SK-OV-3) of human B2M-siRNA or C-siRNA, respectively, and incubated for 24 h. Cell proliferation was measured using the Cell Proliferation Reagent (WST-1) kit (Roche, Cat# 11644807001) according to the manufacturer’s instruction. The signal in OD was read at 450 nm by a plate reader (BioTek Epoch, Winooski, VT, USA). Experiment was repeated at least three times.

### Wound healing assay

Cells were seeded into 6-well plates at a density of 3 × 10^5^ cells/well and cultured up to 85 % confluence. The cell monolayer was then scraped using a pipette tip to generate scratch wounds. After washing three times to remove cell debris, the cells were transfected with B2M-siRNA or C-siRNA and incubated for 24, 48 and 72 h. Images were obtained at different time points and the widths of the gaps were measured and analyzed.

### Cell invasion assay

The Matrigel (250 µg/ml final concentration, BD Biosciences, Cat# 356234, Bedford, MA, USA) was coated on the top chamber of Transwell (pore size 8 μm, Costar, Corning, Cat# 3422, New York, NY, USA) in a 24-well plate. After the solidification of the Matrigel, B2M-siRNA-transfected or C-siRNA-transfected cells (1 × 10^4^ cells, 24-hour post-transfection) were plated in the top chamber of Transwell with 180 μl serum-free medium. The bottom chamber was supplemented with 600 μl medium supplemented with 10 % FBS as a chemo-attractant. After incubation at 37 °C for 48 h, the non-invaded cells were removed from the upper chamber. The invaded cells that had passed through the Matrigel-coated membrane and attached to the lower surface of the membrane were fixed with 4 % paraformaldehyde, stained with 5 % Crystal Violet Staining Solution (Beyotime, Cat# C0121) for visualization, and photographed with a camera. The cell number was counted in three random fields viewed by a light microscope. The tests were repeated at least three times.

### RNA extraction and quantitative real time PCR

Total RNA in the cells was extracted using Trizol reagent (Invitrogen, Thermo Fisher Scientific, Rockford, IL, USA), according to the manufacturer’s instruction. Five-hundred nanogram of total RNA was reversely transcribed using a reverse transcription kit (TaKaRa Biotechnology Co., Ltd., Dalian, Liaoning, China). The primer sequences of human B2M were 5′-GGTTTCATCCATCCGACATTG-3′ (forward) and 5′-CATGTCTCGATCCCACTTAAC-3′ (reverse). The primer sequences of human β-actin were 5′-ACAATGTGGCCGAGGACTTT-3′ (forward) and 5′-GCACGAAGGCTCATCATTCA-3′ (reverse) (synthesized by Sangon Biotech Co., Ltd., Shanghai, China). PCR amplification was performed at 95 °C for 5 s and 60 °C for 30 s for 40 cycles using a SYBR Premix TaqTM II (Tli RNaseH Plus) kit (TaKaRa Biotechnology Co., Ltd.) with an initial step of denaturing RNA at 95 °C for 30 s. Assays were conducted in triplicate and repeated at least three times. The amount of target (B2M) normalized to an endogenous control (β-actin) given by 2^ΔΔCt^, in which threshold cycle (Ct) was obtained using Sequence Detection Software v1.4 (7300 Real Time PCR System, Applied Biosystems, Foster City, CA, USA).

### Western blotting

OVCAR-3 and SK-OV-3 cells were lysed in RIPA lysis buffer (Thermo Scientific, Rockford, USA) with 1 % PMSF (Beyotime) and 1 % phosphatase inhibitor (KeyGEN), followed by sonication. The cell lysates were centrifuged at 14,000 rpm for 15 min at 4 °C. Equal amount proteins were separated on 15 % SDS-PAGE with 4X Protein SDS PAGE Loading Buffer (TaKaRa) and transferred to a PVDF membrane (Millipore, Billerica, MA, USA). After blocking with 5 % non-fat milk in Tris-buffered saline with Tween 20 (TBS-T) for 1 h, the membrane was incubated with a primary antibody at 4 °C overnight and subsequently incubated with horseradish peroxidase-conjugated goat anti-rabbit or anti-mouse IgG (1:10,000 dilution, Cell Signaling Technology, Inc., Danvers, MA, USA) for 1 h at room temperature. The following primary antibodies were used: rabbit anti-B2M (1:3000 dilution, Abcam), mouse anti-Smad2 and rabbit anti-phospho-Smad2 (both 1:2000 dilution, Cat# 3103 and #3101, Cell Signaling Technology, Inc.) and rabbit anti-β-actin (1:5000 dilution, Cat# 66009-1-Ig, Proteintech, Wuhan, China). Signals were detected using Immobilon™ Western Chemiluminescent HRP Substrate (Millipore) and quantified using Tanon-4500 Gel Imaging System with GIS ID Analysis Software v4.1.5 (Tanon Science and Technology Co., Ltd., Shanghai, China).

### Enzyme-linked immunosorbent assay

Cells were seeded into 6-well plates at a density of 2 × 10^5^ cells/well (OVCAR-3) or 1.5 × 10^5^ cells/well (SK-OV-3) and cultured with complete medium for 24 h. After washing with PBS, cells were incubated in serum-free medium without or with 10 ng/ml of TGF-β1 for 24, 48 and 72 h. The culture media were collected and centrifuged at 2000 rpm for 10 min at 4 °C. The cell lysates were extracted as described previously in Western blotting. Equal amount samples were subjected to the enzyme-linked immunosorbent assay (ELISA). The concentration of B2M in medium and cytosol was determined using a Human B2M ELISA Kit (RayBiotech, Cat# ELH-B2M, Norcross, GA, USA) according to the manufacturer’s instruction. Briefly, 100 µl standards and samples were respectively added into the wells and incubated at 4 °C overnight. After washing away any unbound substance, 100 µl biotinylated anti-Human B2M was added into each well and incubated at room temperature for 1 h. After washing, 100 µl HRP-Streptavidin solution was added to each well and incubated at room temperature for 45 min. After another washing, 100 µl TMB One-Step Substrate Reagent was added into each well and incubated at room temperature in the dark for 20 min. After adding 50 µl Stop Solution into each well, the signal in OD was determined at 450 nm using a microplate reader immediately (BioTek Epoch).

### Statistical analyses

All analyses were performed with SPSS Statistics 21 for Windows (SPSS, Chicago, IL, USA). For comparison between two groups of positivity and the association of B2M protein expression with histological types or the clinicopathological characteristics, Chi square test or Fisher’s exact test was applied. For comparison between two groups in treatment experiment, two-sample Wilcoxon rank-sum test was used. Results are presented as the mean ± standard error of mean (SEM). A P value <0.05 was considered to be significant.

## Results

### B2M expression in the epithelial-type tumours of the ovary

**Fig. 1:**
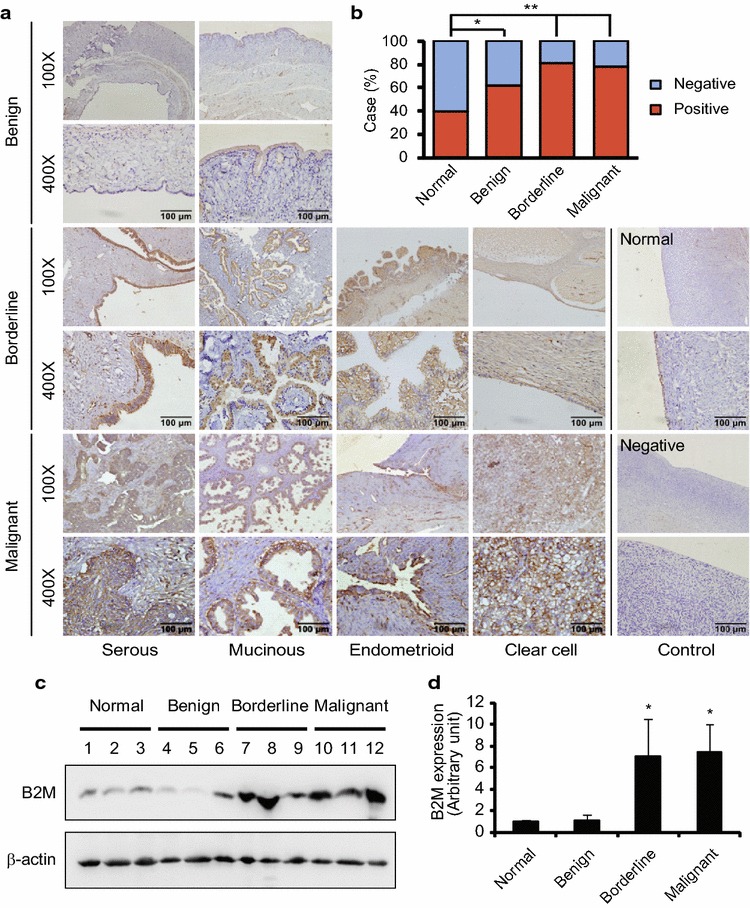
B2M protein expression in human ovarian tissues. **a** Immunohistochemical staining of B2M protein in human epithelia-type ovarian tumours. A *brown color* in epithelial cells is considered as a positive staining. Negative control without first antibody is performed in the normal ovarian tissue. Representative images of B2M expression in serous, mucinous, endometrioid and clear cell tumours and the normal ovarian tissue are shown. Original magnification × 100 and × 400. *Scale bar* 100 µm. **b** The case rate of B2M positive and negative. Positive vs. negative: 16/24 in control without tumour (40 cases), 24/14 in benign tumour (38 cases), 31/7 in borderline tumour (38 cases) and 25/7 in malignant tumour (32 cases). For comparison between two groups, χ^2^ test was applied. **c** Detection of B2M expression in the normal ovarian tissues (case #1–3) and serous benign (case #4–6), borderline (case #7–9) and malignant (case # 10–12) tumours by Western blot. **d** Semi-quantitative analysis after densitometry on the gels of (**c**). Benign, benign tumour; Borderline, borderline tumour; Malignant, malignant tumour; Normal, normal ovarian tissue. *P < 0.05; **P < 0.01

To examine the expression of B2M in ovarian tissue derived from patients with or without ovarian tumour, IHC staining and Western blot were applied. We found that the immunoreactive staining of B2M was observed in ovarian epithelial cells (Fig. 1a). The high staining of B2M was shown in borderline and malignant tumours, including serous, mucinous, endometrioid and clear cell tumours. The case rate of positive expression was significantly increased in patients with benign, borderline, and malignant tumours compared to patients without ovarian tumours (normal vs. benign, P = 0.041; normal vs. borderline, P < 0.001; normal vs. malignant, P = 0.001; benign vs. borderline, P = 0.073; benign vs. malignant, P = 0.173; borderline vs. malignant, P = 0.719) (Fig. 1b). Western blot analysis showed that the expression of B2M was significant higher in the serous borderline and malignant tumours of the ovary (Fig. 1c, d).

### Association of B2M expression with the clinicopathological features of patients with ovarian tumours

Next we examined whether the expression of B2M is associated with the clinicopathological features of patients with epithelial-type ovarian tumours. We observed that the expression of B2M was not associated with the age at the time of diagnosis and with primary tumour size (Table 1). By multiple comparisons of B2M protein expression associated with histopathological characteristics, we found that the positivity of B2M expression was not significantly different between various histological types in benign, borderline and malignant tumours (P > 0.05). There was no significant difference between high-grade and low-grade serous malignant tumours (P = 0.83). Further analysis of patients with EOC showed that the expression of B2M had no association with clinicopathological features, such as age (≤45 vs. >45), tumour size (≤2 cm vs. >2 cm), multifocal tumours, lymph node metastasis, and clinical stages (Table 2) (all P > 0.05).

**Table 1:** Association of B2M expression with the clinico- and histo-pathological features of patients with epithelial-type ovarian tumours

Clinicopathological features	n	B2M expression		P value
		Positive n (%)	Negative n (%)	
Age at diagnosis				
≤45	41	32 (78.05)	9 (21.95)	0.567
>45	67	49 (73.13)	18 (26.87)	
Primary tumour size				
≤2 cm	7	6 (85.71)	1 (14.29)	0.498
>2 cm	101	75 (74.26)	26 (25.74)	
Benign tumour				
Serous	16	12 (68.75)	4 (31.25)	0.326^a^
Mucinous	18	9 (88.24)	9 (11.76)	
Endometrioid	4	2 (50.00)	2 50.00)	
Borderline tumour				
Serous	14	10 (71.43)	4 (28.57)	0.191^b^
Mucinous	21	19 (90.48)	2 (9.52)	
Clear cell	1	1 (100)	0	
Endometrioid	1	1 (100)	0	
Transitional cell	1	1 (100)	0	
Malignant tumour				>0.999^c^
Serous				
High-grade	12	11 (91.67)	1 (8.33)	0.083^d^
Low-grade	6	3 (50.00)	3 (50.00)	
Mucinous	5	4 (80.00)	1 (20.00)	
Clear cell	7	5 (71.43)	2 (28.57)	
Endometrioid	1	1 (100)	0	
Transitional cell	1	1 (100)	0	

The expression of B2M protein was detected by immunohistochemistry. For comparison of B2M expression associated with age, χ^2^ test was applied. For comparison of B2M expression associated with primary tumour size, χ^2^ test with continuity correction was applied. For multiple comparisons of B2M expression associated with the histopathological features, Fisher’s exact test was applied

*n* number of cases; *Positive* positive expression; *Negative* negative expression

^a^Multiple comparisons of the histological types (serous, mucinous and endometrioid tumours) in benign tumors

^b^Comparison between serous and mucinous borderline tumours

^c^Multiple comparisons of the histological types (serous, mucinous and clear cell tumours) in malignant tumors

^d^Comparison between low-grade serous tumour and high-grade serous tumour

**Table 2:** Association of B2M expression with the clinicopathological features of patients with EOC

Clinicopathological features	n	B2M expression		P value
		Positive (%)	Negative (%)	
Age at diagnosis				
≤45	8	6 (75.00)	2 (25.00)	>0.999
>45	24	19 (79.17)	5 (20.83)	
Primary tumour size				
≤2 cm	4	4 (100.00)	0 (0.00)	0.552
>2 cm	28	21 (75.00)	7 (25.00)	
Multifocal tumours				
Yes	15	13 (86.67)	2 (13.33)	0.402
No	17	12 (70.59)	5 (29.41)	
LN metastasis				
Yes	4	3 (75.00)	1 (25.00)	>0.999
No	28	22 (78.57)	6 (21.43)	
FIGO stage				
I	17	12 (70.59)	5 (29.41)	0.207
II	7	5 (71.43)	2 (28.57)	
III	8	8 (100.00)	0 (0.00)	

The expression of B2M proteins was detected by immunohistochemistry. For comparison of B2M expression associated with age, primary tumour size, multifocal, LN metastasis, and clinical stages, Fisher’s exact test was applied

*EOC* epithelial ovarian cancer; *n* number of cases; *Positive* positive expression; *Negative* negative expression; *LN* lymph node

### Effect of knocking down of B2M on ovarian cancer cell proliferation, migration and invasion

**Fig. 2:**
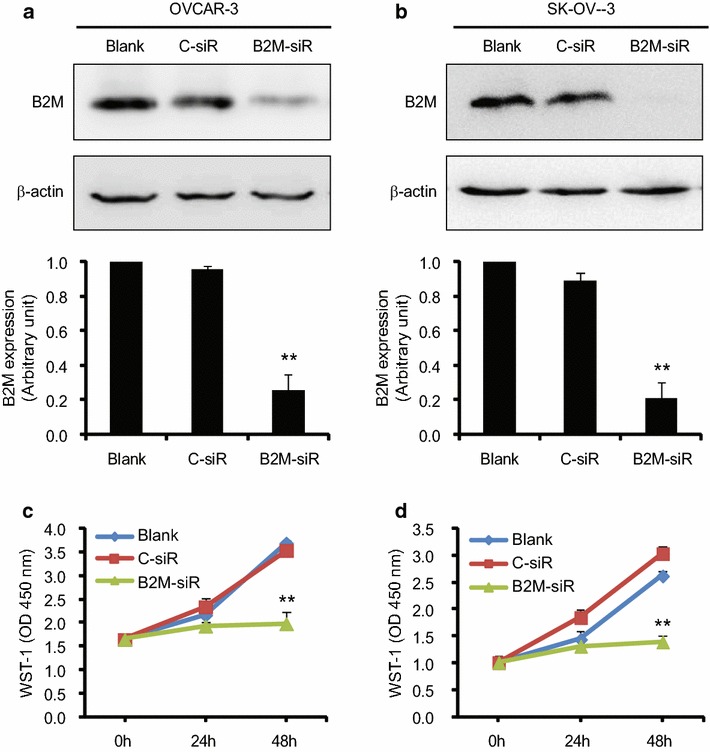
Measurement of cell proliferation. Transfection efficiency in OVCAR-3 (**a**) and SK-OV-3 (**b**) cells. A knocking down of B2M was detected by Western blot (*top panel*), followed by the densitometry of the gel (n = 3). The cell proliferation of OVCAR-3 (**c**) and SK-OV-3 (**d**) was determined by the WST-1 assay after siRNA transfection (n = 3). The results are representative of three independent experiments. Blank, control without transfection; C-siR, control siRNA; B2M-siR, B2M-siRNA. **P < 0.01

To determine the functional effects of B2M on the biological behaviors of OC cells, a loss-of-function approach was applied. First, several siRNAs specific to human B2M (B2M-siRNA) were synthesized. Second, transfection efficiency was tested prior to full experiments. Reduction of B2M protein was confirmed in two EOC cell lines, OVCAR-3 and SK-OV-3, by Western blot (Fig. 2a, b). Next we examined cell proliferation by WST-1 assay. The inhibition of B2M expression by B2M-siRNA significantly decreased OVCAR-3 and SK-OV-3 cell proliferation at 48 h post-transfection (Fig. 2c, d; n = 3; P < 0.01).

**Fig. 3:**
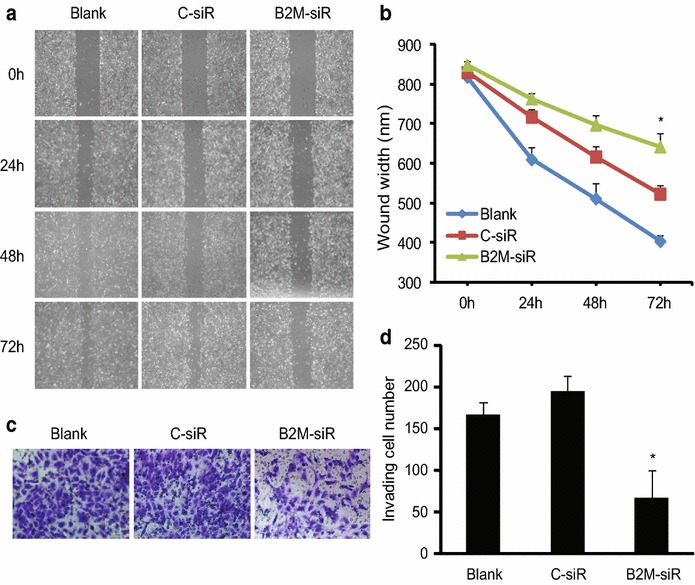
Migration and invasion of SK-OV-3 cells. **a** Migration of SK-OV-3 after transfection. A wound healing assay was performed and compared between the blank, negative control and B2M-siRNA transfected cells. The photos of wounds were taken at different times. Original magnification × 100. **b** The quantitative analysis of the wound width was shown in the *line chart*. Three independent experiments were conducted and similar results were obtained. **c** Invasion of SK-OV-3 after transfection. The invasive property of the cells was determined at 48 h by invasion assays using Matrigel invasion chambers and the photos were taken. Original magnification × 200. **d** Quantification of invading cells. The results are *plotted* as the average number of invading cells from three random microscopic fields. Three independent experiments were performed and similar results were obtained. *P < 0.05

To evaluate the effect of B2M on OC cell migration, a wound-healing assay was performed in SK-OV-3 cells. As shown in Fig. 3a, b, the suppression of B2M expression significantly inhibited SK-OV-3 cell migration compared with the blank and negative control (C-siR) (n = 3; P < 0.05).

Next we examined the effect of B2M on cell invasion in SK-OV-3 cells. After culture for 48 h, the non-invading cells at the upper layer of chamber were removed and the number of invading cells at the low layer was counted. We found that the suppression of B2M expression significantly inhibited SK-OV-3 cell invasion by approximately 65 % (Fig. 3c, d; n = 3; P < 0.05).

### Regulation of B2M expression by the TGF-β signaling pathway in ovarian cancer cells

**Fig. 4:**
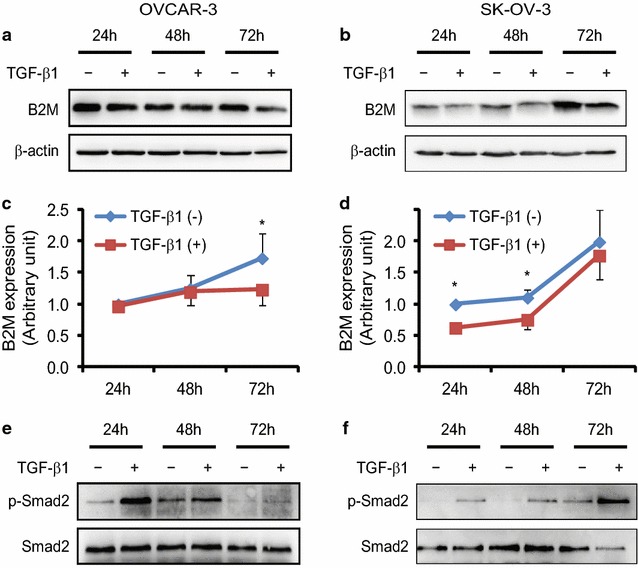
Effect of TGF-β1 on the expression of B2M protein in ovarian cancer cell lines. In a time-course study, OVCAR-3 (**a**) and SK-OV-3 (**b**) cells were treated with 10 ng/ml of TGF-β1 for 24, 48 and 72 h, respectively. Equal amounts of total protein were subjected to SDS-PAGE and transferred to a PVDF membrane. Specific signal was detected by Western blot analysis using a specific antibody against B2M or β-actin. **c**, **d** The *graphs* show the quantitative analysis of the gels from OVCAR-3 and SK-OV-3 cells, respectively, after densitometry (both n = 3). β-actin was served as a loading control. *P < 0.05. Phospho-Smad2 (p-Smad2) and total Smad2 were used as indicators for the TGF-β signaling pathway existed in those cells. p-Smad2 was increase upon TGF-β1 stimulation in OVCAR-3 (**e**) and SK-OV-3 (**f**) cells

Since TGF-β signaling pathway plays a significant role in ovarian tumorigenesis and B2M was associated with the biological behaviour of OC, we subsequently investigated whether the TGF-β signaling pathway mediates B2M expression. OVCAR-3 and SK-OV-3 cells were treated with TGF-β1 (10 ng/ml) for 24, 48 and 72 h, respectively. By Western blot analysis we observed that B2M expression, after normalized to β-actin, was significantly decreased after TGF-β1 treatment at 72 h in OVCAR-3 cells (Fig. 4a, c; P < 0.05). We also found a decrease of B2M expression in SK-OV-3 after TGF-β1 treatment for 24 and 48 h (Fig. 4b, d; P < 0.05). Because Smad2 is a TGF-β signaling transducer protein and is activated upon TGF-β1 treatment, we subsequently detected the phosphorylation of Smad2 to see if these cells respond to TGF-β1. Indeed, an increase of Smad2 phosphorylation was observed in TGF-β1 treated OVCAR-3 (Fig. 4e) and SK-OV-3 (Fig. 4f) cells, indicating that the TGF-β signaling pathway exists in those cells.

**Fig. 5:**
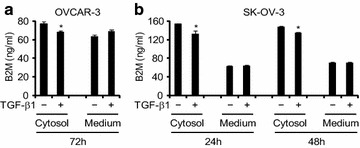
Measurement of B2M concentration after TGF-β1 treatment. The concentration of B2M in the cytosol and culture medium of OVCAR-3 and SK-OV-3 cells in the absence (−) or presence (+) of 10 ng/ml TGF-β1 for 72 h (**a**) and 24 and 48 h (**b**), respectively, was determined by the ELISA assay. *P < 0.05

The suppression of B2M by TGF-β1 was further confirmed by an ELISA assay. We found that TGF-β1 (10 ng/ml) significantly decreased cytosolic B2M in OVCAR-3 cells after 72 h treatment and in SK-OV-3 cells after 24 and 48 h treatment, respectively, but the concentration of B2M in the culture medium was not affected (Fig. 5a, b).

**Fig. 6:**
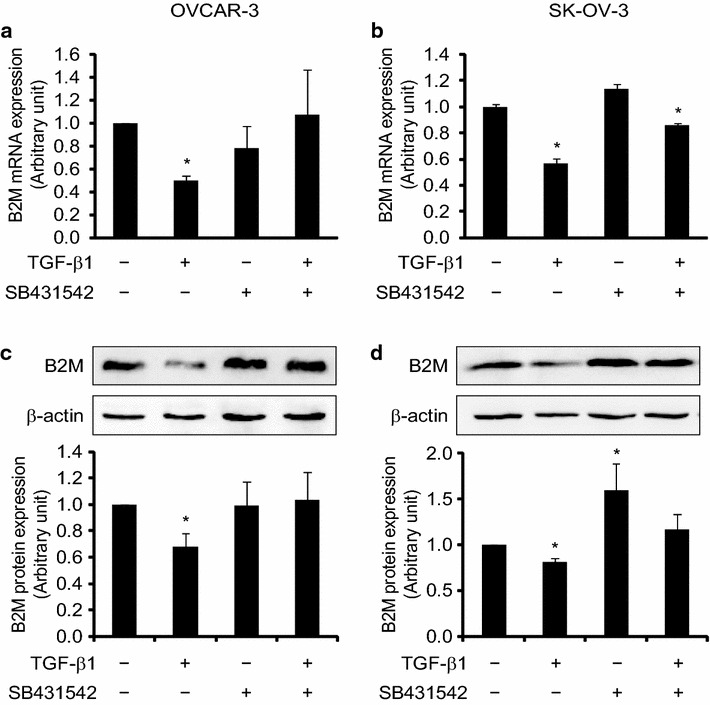
Regulation of the expression of B2M at mRNA and protein levels by the TGF-β signaling pathway in ovarian cancer cell lines. OVCAR-3 and SK-OV-3 cells were pre-treated with a TGF-β type I receptor kinases inhibitor (SB431542, 10 μM) for 30 min and then treated with 10 ng/ml TGF-β1. B2M mRNA in OVCAR-3 (**a**) and SK-OV-3 (**b**) cells was detected by quantitative RT-PCR using primers specific to B2M after TGF-β1 treatment for 24 h. B2M protein in OVCAR-3 (**c**) and SK-OV-3 (**d**) cells was detected by Western blot analysis using a specific antibody against B2M and β-actin after TGF-β1 treatment for 72 and 48 h, respectively. Histograms show the quantitative analysis of the gels after densitometry. The results are representative of three independent experiments. *P < 0.05

Next, we blocked the TGF-β signaling pathway by the inhibitor of TGF-β type I receptor kinases, SB-431542, to evaluate whether the B2M expression is mediated by the TGF-β signaling pathway. By comparing with the vehicle control group, we found that TGF-β1 had an inhibitory effect on the expression of B2M. The expression of B2M mRNA was significantly decreased after 10 ng/ml TGF-β1 treatment for 24 h in OVCAR-3 (Fig. 6a) and SK-OV-3 (Fig. 6b) cells detected by quantitative RT-PCR (both P < 0.05). This downregulation of B2M mRNA expression by TGF-β1 was attenuated in the cells pre-treated with 10 μM SB-431542 for 30 min. The decrease of B2M expression was also observed at the protein level detected by Western blot. We found that the expression of B2M protein was significantly decreased in OVCAR-3 (Fig. 6c) and SK-OV-3 (Fig. 6d) cells after 10 ng/ml TGF-β1 treatment for 72 and 48 h, respectively, and the decrease of B2M by TGF-β1 was abolished in the presence of SB-431542 (P < 0.05). These data indicate that the expression of B2M is regulated by the TGF-β signaling pathway.

## Discussion

The current study showed the overexpression of B2M in human epithelial-type borderline and malignant tumours and evaluated the expression of B2M associated with clinicopathological features in patients with EOC. Using a loss-of-function approach, the biological function of B2M in OC cells was examined. Furthermore, we explored for the first time the regulation of B2M expression at mRNA and protein levels by the TGF-β signaling pathway in OC cells.

B2M is a component of the MHC class I complex associated with the α-chain of the complex to regulate the immune system in human. It has been shown that B2M is a biomarker of inflammation, while the inflammation is associated with cancers. Indeed, more and more evidences provide the information that B2M plays a role in cancer biology. Clinical studies suggest that soluble B2M is a serum marker of the several types of cancers, including OC. In early 1979, the high level of serum B2M is found in 56 % OC patients [9]. Recently, an increase of B2M concentration is also detected in saliva [26] and peritoneal fluid [27] of patients with OC. Data mining of NCBI GEO DataSets showed that B2M mRNA was expressed in OC epithelial cells as well as in ovarian normal surface epithelia cells in the study of gene expression profiling analyses [28]. However, the expression of B2M in the different types of ovarian tumours is barely reported. Current study examined for the first time the expression of B2M in human different epithelial-type ovarian tumours compared with the normal ovary. The positivity of B2M expression was higher in patients with ovarian tumour than in patients without tumour. The highest expression of B2M was found in patients with borderline and malignant tumours compared with women with normal ovary, suggesting that the overexpression of B2M may be associated with aggressive disease. Current study did not test the serum level of B2M due to the lack of serum collection before or after surgery, but previous study has shown that the serum and peritoneal fluid of B2M was significant higher in patients with OC than in patients with benign neoplasms [27]. Our further analysis of B2M expression in different histopathological types of ovarian tumours showed that B2M protein expression was not associated with clinical features, such as age, tumour size, multifocal tumours, lymph node metastasis and clinical stage. Although the number of cases in this study is relatively small (total 108 patients with ovarian tumour), our findings are similar to the results obtained from a breast cancer study that the B2M expression has no significant association with age, lymph node metastasis and clinical stage [29]. However in patients with gallbladder cancer, B2M expression is significantly correlated with large tumour size, high TNM stage, lymph node metastasis and invasion of carcinomas [30]. These data suggest that the association of B2M expression with clinical features is tumour-type specific. Nevertheless, our findings, coming together with recent evidences from other laboratories, support the notion that B2M is a potential marker for the diagnosis of OC, especially the epithelial-type borderline and malignant tumours of the ovary.

It has been shown that the overexpression of B2M promotes SK-OV-3 cell proliferation and an antibody against B2M inhibits proliferation and induces apoptosis [31]. Besides, B2M is also involved in promoting lethal bone and soft tissue metastases in host mice [32]. Our in vitro studies demonstrate that knocking down of B2M leads to the decrease of OC cell growth, as well as their migration and invasion, indicating that B2M has a cellular function in regulating cell behaviors, such as cell proliferation, migration and invasion. These data suggest that B2M may play an important role in the development of OC.

Ovarian tumorigenesis is mediated by various signaling pathways, including the TGF-β signaling pathway. Our recent study has shown that TGF-β1 regulates cystatin B, a progression marker of human epithelial ovarian tumour, in OC cells [25]. Studies from other groups also show that TGF-β modulates serous borderline ovarian tumour invasion [23] and increased the metastatic potential of OC cells [24]. However, whether the TGF-β signaling pathway involved in B2M regulation remains unknown. The current study examined for the first time that TGF-β1 mediates B2M expression at mRNA and protein levels in OC cells. A downregulation of B2M expression was observed after TGF-β1 treatment in OVCAR-3 and SK-OV-3 cells. The inhibition of TGF-β signaling by its type I receptor inhibitor abolished the downregulation of B2M, indicating that the expression of B2M is indeed regulated by the TGF-β signaling pathway. However, how TGF-β regulates B2M expression in vivo is not completely understood. The data from public database NCBI GEO DataSets reveal that TGF-β1 did not affect B2M expression within 12 h in an immortalized ovarian surface epithelial (IOSE) cell line in a time-course study as there is no direct evidence showing a Smad specific target promoter in B2M gene [33]. We found that TGF-β1-mediated B2M expression needs at least 24 h, indicating that TGF-β1 regulates B2M expression via other mechanisms. The dysregulation of TGF-β signaling during OC development [34, 35] may lead to the upregulation of B2M expression. Indeed, the alteration of TGF-β components is found in human OC [36–39]. Further study in an animal model of OC is needed to examine the molecular mechanism underlying TGF-β-mediated B2M expression.

Because B2M is non-covalently linked to the α heavy chain of MHC class I complex and the loss of MHC class I molecules is one of the mechanisms for tumours to escape the recognition and destruction of immune system, the dysregulation of B2M between the membrane and soluble forms may play a role in ovarian tumorigenesis. Although we detected a reduction of cytosolic B2M by TGF-β1, the level of B2M in the culture medium was not significantly changed due to the larger volume compared to the cytosolic volume. Previous studies have shown that OC cell lines express MHC class I molecules as well as immunosuppressive TGF-β1 mRNA and protein which was only modestly detected in cell culture supernatants [40, 41]. The poor secretion of TGF-β1 in tumour cells may result in low autocrine and exocrine of this cytokine and finally lead to the induction of B2M expression. The current study examined the expression of B2M in ovarian tumours, but whether B2M is involved in tumour cell immune-escape in addition to immunosurveillance in patients with OC will need to be evaluated in subsequent studies.

## Conclusions

B2M was overexpressed in human epithelial-type borderline and malignant tumours of the ovary. B2M acted as a positive regulator in the proliferation, migration and invasion of OC cells and was regulated at mRNA and protein levels by the TGF-β signaling pathway, indicating a possible role of B2M in ovarian tumorigenesis. Targeting B2M, such as using siRNA, may provide a potential therapeutic application for patients with B2M-overexpressing ovarian tumour.

## Abbreviations

B2M: beta-2 microglobulin
ELISA: enzyme-linked immunosorbent assay
EOC: epithelial ovarian cancer
IHC: immunohistochemistry
MHC: major histocompatibility complex
OC: ovarian cancer
TGF-β: transforming growth factor-β
